# Comparison of various indices for predicting sarcopenia and its components in patients receiving peritoneal dialysis

**DOI:** 10.1038/s41598-022-18492-2

**Published:** 2022-08-18

**Authors:** Jun Young Do, Seok Hui Kang

**Affiliations:** Department of Internal Medicine, College of Medicine, Yeungnam University, 170 Hyeonchung-ro, Nam-gu, Daegu, 42415 Republic of Korea

**Keywords:** Diseases, Medical research, Nephrology, Risk factors

## Abstract

This study aimed to evaluate and compare the usefulness of four indices—arm circumference, thigh circumference, mid-arm muscle circumference (MAMC), and thigh muscle circumference (TMC)—with that of other classical indicators of body composition in the prediction of sarcopenia and two sarcopenia-related components in patients receiving peritoneal dialysis (PD) grouped by sex. The data of all patients receiving PD who visited a tertiary medical center were collected (n = 214); of them 199 patients undergoing PD were included in the final analyses. Data on baseline characteristics and measurements, including circumferences of appendicular sites, handgrip strength (HGS), and appendicular lean mass (ALM) index, were obtained during a routine peritoneal membrane equilibration test. Body composition was evaluated using dual-energy X-ray absorptiometry. The ALM index (kg/m^2^) was defined as the sum of lean mass in the upper and lower extremities divided by height squared. Sarcopenia was defined as low HGS and low muscle mass based on the cut-off values in the Asian Working Group for Sarcopenia guideline. The circumferences of the lower extremities showed the greatest association with the ALM index in both sexes. Prediction of HGS was better with the MAMC than with the other indices in the male patients, whereas none of the indices were associated with HGS in the female patients. Moreover, the MAMC in the male patients and TMC in the female patients were the strongest predictors of sarcopenia among the six anthropometric indices. This study showed that the MAMC in male PD patients and TMC in female PD patients might be the best predictors of sarcopenia. However, the TMC was associated with sarcopenia regardless of HGS in the female PD patients. These findings suggest that, in PD patients, different indices should be considered in predicting sarcopenia or its components based on the sex.

## Introduction

Peritoneal dialysis (PD) is an important dialysis modality in patients with kidney failure who require kidney replacement therapy. However, improvement in the survival of patients receiving PD is associated with an increase in chronic complications^1^. Sarcopenia is an important complication in these patients, possibly associated with aging or accelerated aging processes owing to chronic exposure to uremic toxins, dialysis treatment, or comorbidities^2^. Previous studies in the general populations have shown a prevalence of sarcopenia of 0.8 and 10.5% in older adults^3,4^. Although different diagnostic criteria were used among studies, the prevalence of sarcopenia in patients receiving PD was estimated at 4.0–48% in Asian populations and 11–47.2% in European or US populations^5^. The development of sarcopenia is associated not only with poor clinical outcomes and quality of life, but also with increased health-care burden in patients receiving PD^2^. Furthermore, patients with physical dysfunction due to sarcopenia may require hemodialysis, despite being medically indicated for PD^6^. These clinical implications have led to increased interest among clinicians in the diagnosis and treatment of sarcopenia in patients receiving PD.

A definite diagnosis of sarcopenia ideally requires muscle mass measurements using dual energy x-ray absorptiometry (DXA) or bioimpedance analysis and strength measurement using handgrip strength (HGS) assessment^7^. However, these measurements certain tools and are time consuming. Moreover, the routine use of these two methods in clinical practice is difficult. Therefore, proper screening methods would be useful in identifying patients receiving PD who require further evaluation for diagnosing sarcopenia. Measuring the circumferences of appendicular sites in the body may be an easy and cheap option for predicting sarcopenia. Calf circumference measurement is one of the methods used for screening sarcopenia. Previous studies have shown that using calf circumference measurements and questionnaires is very useful in screening for sarcopenia in the general population^8,9^. However, patients receiving PD have a greater proportion of fat mass (FM) and volume overloading than the general population. Overestimation of the calf circumference owing to volume overload can limit the accuracy of the measurement. The arm circumference (AC), thigh circumference (TC), mid-arm muscle circumference (MAMC), and thigh muscle circumference (TMC) have also been used to determine the nutritional status of patients receiving dialysis, and these can be used to estimate the muscle mass of patients. These indices would be less influenced by the volume status than the calf circumference, however, their usefulness in predicting sarcopenia has not been evaluated fully. This study aimed to evaluate and compare the usefulness of four indices—AC, TC, MAMC, and TMC—with that of other classical indicators of body composition in the prediction of sarcopenia and two sarcopenia-related components in patients receiving PD grouped by sex.

## Methods

### Study population

This retrospective, cross-sectional study re-analyzed a dataset from a previous study^10^. Briefly, we included all patients receiving PD who met the following criteria: age of ≥ 20 years, ability to communicate, and no hospitalization within the last 3 months except for PD catheter insertion. Meanwhile, we excluded the patients who met the following criteria: inability to ambulate, amputated limb, liver disease, severe cardiopulmonary disease, acute or chronic musculoskeletal disorders, or any neurological or psychiatric disturbances. The data of all patients receiving PD who visited a tertiary medical center between September 2017 and November 2020 were collected (n = 214), and informed consent was obtained from all of them. Data on baseline characteristics and measurements including circumferences of appendicular sites (AC, TC, MAMC, and TMC), HGS, and appendicular lean mass (ALM) index, were obtained during a routine peritoneal membrane equilibration test within the study period. This study was approved by the Institutional Review Board of the Yeungnam University Medical center and was conducted in accordance with the principles of the World Medical Association’s Declaration of Helsinki (approval no: 2020–06-002).

### Baseline variables

We collected baseline data on age, sex, presence of diabetes mellitus (DM), use of automated PD, dialysis vintage (interval between the initiation of PD and the time of study evaluation [months]), weekly Kt/V_urea_, C-reactive protein (CRP) level (mg/dL), dialysate per serum creatinine level at 4 h (DP4Cr) ratio, urine volume (mL/day), and serum calcium (mg/dL), phosphorus (mg/dL), sodium (mmol/L), potassium (mmol/L), and albumin (g/dL) levels. All laboratory studies were performed after overnight fasting. DM was defined based on a patient-reported history of DM, diagnosis from medical records, or use of DM medications. The DP4Cr ratio was calculated during the peritoneal membrane equilibration test, while the weekly Kt/V_urea_ was calculated using 24-h urine and dialysate collections as described previously^11^. The weekly Kt/V_urea_ is an indicator of dialysis adequacy or dose. Generally, an inadequate dialysis dose is associated with retained uremic toxins, which can lead to the development of malnutrition. Meanwhile, the DP4Cr is an indicator of peritoneal membrane characteristics. Generally, patients with a high DP4Cr are prone to developing volume overload through rapid absorption of glucose and malnutrition through peritoneal protein loss. These indicators can be associated with malnutrition or sarcopenia in patients receiving PD; thus, we evaluated these indicators herein.

### Body composition, strength, and body circumference assessment

Body compositions were evaluated using DXA (Hologic, Madison, WI, USA). The measurements were performed after dialysate drainage, with the patients in the supine position and wearing a light gown. Lean mass (LM) and FM were also measured using DXA. The ALM index (kg/m^2^) was defined as the sum of LM in the upper and lower extremities divided by height squared. The average LM and FM of the arms or legs on both sides were used. HGS was measured in all patients using a digital dynamometer (Takei 5401; Takei Scientific Instruments Co., Ltd, Niigata, Japan). Each patient underwent three trials with the dominant hand; the highest value among the three trials was considered the HGS.

Sarcopenia was defined as low HGS and muscle mass according to the Asian Working Group for Sarcopenia guideline^7^. In the guideline, a low HGS is defined as an HGS of < 28 kg for men and < 18 kg for women, and a low muscle mass as an ALM index of < 7.0 kg/m^2^ for men and < 5.4 kg/m^2^ for women.

We evaluated the following six indices of body size: body mass index (BMI), waist circumference (WC), AC, TC, MAMC, and TMC. The BMI was calculated by dividing the body weight by the height squared (kg/m^2^). The WC, AC, TC, MAMC, and TMC were measured using previous protocols^12–17^. These measurements were obtained after peritoneal dialysate drainage. All measurements were conducted by a trained nurse. For measuring the WC, the start of the measuring tape was placed at the lower margin of the last palpable rib and extended down the mid-axillary line to the top of the hipbone. A vertical mark was made at the midpoint, and this mark was crossed with a perpendicular line. The tape was positioned perpendicular to the long axis of the trunk at the marked point, ensuring that the tape was wrapped over the same spot on the opposite side. The measurement was recorded at the end of a normal expiration.

The AC was measured at the midpoint between the tip of the shoulder and the elbow on the non-dominant side of the body using a flexible and non-stretchable tape; the measurement was read to the nearest 0.1 cm. The thickness of the triceps skin fold was measured using a skinfold caliper and read to the nearest 0.2 cm. The MAMC was calculated as follows: MAMC (cm) = AC (cm) − (π × triceps skin fold thickness [cm]).

For measuring the TC, the measuring tape was placed horizontally around the thigh, midway between the midpoint of the inguinal crease and the proximal border of the patella. The measurement was made with the tape touching the skin around the entire circumference but without compressing the soft tissues. The mid-thigh skin fold thickness was measured on the anterior aspect of the measured point of the TC using a skinfold caliper and read to the nearest 0.2 cm. The TMC was calculated as follows: TMC (cm) = TC (cm) − (π × mid-thigh skin fold thickness [cm]).

### Statistical analysis

The data were analyzed using SAS (version 9.4; SAS, Cary, NC, USA). The categorical variables were expressed as numbers (percentages) and analyzed using Pearson’s χ^2^ or Fisher’s exact test. The distribution of the continuous variables was evaluated using the Kolmogorov–Smirnov test. These variables were presented as means ± standard deviations for normally distributed data and medians (interquartile ranges, 25th–75th) for non-normally distributed data. The continuous variables with a non-normal distribution were compared using the Mann–Whitney U-test and those with a normal distribution using a t-test. The associations between the six indices and other continuous variables were evaluated using Pearson’s correlation, partial correlation, and linear regression analyses. The associations between the six indices and sarcopenia were evaluated using logistic regression analysis. All variables were not adjusted owing to the limited sample size of the study. We selected seven variables with a significant association with the nutritional/inflammatory status and excluded variables with an association with body size in relation to multicollinearity. Age, DM as an important comorbidity, weekly Kt/V_urea_ as an indicator of dialysis adequacy, urine volume as an indicator of residual renal function, serum albumin level as serologic nutritional marker, CRP level as an inflammatory indicator, and use of automated PD as a factor influencing the volume status or residual renal function were included in our analyses. Automated PD consists of approximately 3–5 exchanges of peritoneal dialysate during an 8–12 h period with or without long-term dwell for remaining time. Whereas continuous ambulatory PD requires approximately 3–4 exchanges of peritoneal dialysate in 24 h. Water is removed via both aquaporins and small pores of the peritoneum, but sodium is mainly removed via small pores^18–20^. Therefore, automated PD with only short dwells would be associated with lower sodium removal than continuous ambulatory PD. Patients undergoing automated PD would be prone to more volume overload because of lower sodium removal. Conversely, patients with greater residual renal function may have a tendency of performing automated PD. These variables are well-known indicators with direct or indirect associations with muscle mass or strength. Therefore, the multivariate analyses were adjusted for age, presence of DM, weekly Kt/V_urea_, urine volume, serum albumin level, CRP level, and use of automated PD. The multivariate model was applied using these seven covariates and one of the body indices (BMI, WC, TC, AC, TMC, or MAMC). The variance inflation factor was used to identify multicollinearity for the multivariate regression model. A variance inflation factor greater than 10 was not accepted; nevertheless, the values from all models were < 2.1, and all variables were acceptable. The area under the receiver operating characteristic curve (AUROC) was calculated to determine the ability of the indicators to predict sarcopenia. We further calculated the integrated discrimination improvement (IDI) and net reclassification improvement (NRI) with a category-free option among the models, following the methodology by Pencina et al.^21,22^. *P*-values of < 0.05 were considered statistically significant.

## Results

### Clinical characteristics of participants

Among the 214 participants, 15 were excluded due to missing data (n = 9) or inability to ambulate due to an amputated limb (n = 6). Therefore, 199 patients undergoing PD were included in the final analyses. The baseline characteristics are shown in Table 1. The mean age of the participants was 55.7 ± 12.1 years (55.4 ± 12.5 years for the male patients and 55.7 ± 12.0 years for the female patients; *P* = 0.865). The proportion of patients with DM and those receiving automated PD was greater among the male patients than among the female patients (patients with DM: *P* = 0.003; patients receiving automated PD: *P* = 0.036). The WC, AC, TC, MAMC, and TMC of the male and female participants were 78.1 (72.6–86.6) cm and 73.0 (68.9–80.7) cm; 29.9 (28.3–32.0) cm and 27.7 (25.8–29.4) cm; 51.2 (48.4–55.1) cm and 48.7 (46.8–50.5) cm; 27.4 (26.0–29.2) cm and 24.2 (23.0–25.6) cm; and 47.3 (45.1–51.5) cm and 43.2 (40.6–45.8) cm, respectively (*P* < 0.001 for each). The percentage of the arm LM or FM per sum of the arm LM and FM was 66.6 ± 9.0% and 33.4 ± 9.0% in the male patients and 55.5 ± 11.1% and 44.5 ± 11.1% in the female patients, respectively. The percentage of the leg LM or FM per sum of the leg LM and FM was 72.3 ± 6.4% and 27.7 ± 6.4% in the male patients and 64.2 ± 9.6% and 35.8 ± 9.6% in the female patients, respectively (*P* < 0.001). The prevalence of sarcopenia was 37.7% (n = 75) in the total participants (40.7% [n = 46] in the male patients and 33.7% [n = 29] in the female patients, *P* = 0.314). The number of patients with normal ALM index and normal HGS, normal ALM index and low HGS, low ALM index and normal HGS, and sarcopenia was 24 (21.2%), 12 (10.6%), 31 (27.4%), and 46 (40.7%) for males, and 19 (22.1%), 21 (24.4%), 17 (19.8%), and 29 (33.7%) for females, respectively (*P* = 0.059).

**Table 1:** Clinical characteristics of participants.

Characteristics	Total (n = 199)	Males (n = 113)	Females (n = 86)	*P*-value
Age (years)	55.7 ± 12.1	55.4 ± 12.5	55.7 ± 12.0	0.865
Sex (male)	113 (56.8%)	–	–	–
Diabetes mellitus (%)	98 (49.2%)	66 (58.4%)	32 (37.2%)	0.003
Use of automated peritoneal dialysis (%)	57 (28.6%)	39 (34.5%)	18 (20.9%)	0.036
Dialysis vintage (months)	50 (25–88)	50 (25–76)	64 (26–116)	0.033
Body mass index (kg/m^2^)	24.2 (21.9–26.4)	24.8 (22.4–27.5)	23.7 (21.5–25.2)	0.004
Weekly Kt/V_urea_	1.84 (1.62–2.10)	1.67 (1.50–1.93)	1.99 (1.81–2.23)	< 0.001
C-reactive protein (mg/dL)	0.17 (0.06–0.45)	0.19 (0.08–0.45)	0.12 (0.05–0.45)	0.028
DP4Cr	0.66 ± 0.13	0.68 ± 0.12	0.64 ± 0.15	0.037
Urine volume (mL/day)	50 (0–590)	50 (0–850)	50 (0–400)	0.018
Calcium (mg/dL)	8.3 (7.7–8.8)	8.2 (7.4–8.6)	8.5 (8.0–9.0)	< 0.001
Phosphorus (mg/dL)	4.9 ± 1.4	4.9 ± 1.4	4.9 ± 1.3	0.936
Sodium (mEq/L)	137 (134–139)	137 (134–139)	136 (134–139)	0.910
Potassium (mEq/L)	4.6 ± 0.7	4.5 ± 0.7	4.5 ± 0.7	0.979
Albumin (g/dL)	3.6 ± 0.5	3.6 ± 0.5	3.6 ± 0.4	0.995
Appendicular lean mass index (kg/m^2^)	6.0 (5.2–6.7)	6.6 (5.8–7.3)	5.3 (4.9–6.0)	< 0.001
Handgrip strength (kg)	23.6 ± 8.8	28.4 ± 8.1	17.3 ± 4.8	< 0.001
Arms fat mass (kg)	2.4 (1.8–3.3)	2.4 (1.8–3.3)	2.4 (1.9–3.3)	0.314
Arms lean mass (kg)	4.0 (3.1–4.8)	4.7 (4.0–5.4)	3.1 (2.8–3.4)	< 0.001
Legs fat mass (kg)	5.3 (4.2–6.6)	5.0 (4.3–6.2)	5.4 (4.0–6.6)	0.569
Legs lean mass (kg)	12.1 (9.9–13.8)	13.8 (11.7–15.6)	9.8 (8.9–11.3)	< 0.001

Data are expressed as means ± standard deviations for continuous variables with normal distribution and as medians (interquartile ranges, 25th–75th) for those without normal distribution. Categorical variables are expressed as numbers (percentages). *P*-values were tested between males and females using Mann*–*Whitney U-test for continuous variables with non-normal distribution and *t*-test for those with normal distribution. Categorical data were compared using Pearson’s χ^2^ or Fisher’s exact test.

Abbreviations: DP4Cr, four-hour dialysate-to-plasma creatinine concentration ratio.

### Association between the six indices and FM or LM

The correlation coefficients between the six indices and FM or LM are shown in Table S1. In the male patients, all six indices correlated with the arm FM, arm LM, leg FM, leg LM, ALM index, and HGS. The partial correlation analysis showed that the MAMC had the greatest association with the ALM index among the six indices. The MAMC also had the greatest association with HGS among the six indices. In the female patients, all indices, except for the WC, correlated with the ALM index. Furthermore, the TMC had the greatest association with the ALM index among the six indices. However, there was no significant correlation between the six indices and HGS in the partial correlation analysis.

Table 2 shows the results of the linear regression analyses. The ALM index was associated with the six indices in the male patients and only five indices in the female patients; the TC in the male patients and TMC in the female patients had the greatest standardized-β value among the six indices. The multivariate linear regression analyses showed that the MAMC had the greatest standardized-β value in association with HGS among the six indices in the male patients; however, no association between HGS and the six indices was found in the female patients.

**Table 2:** Results of linear regression analyses of variables for predicting appendicular lean mass index and handgrip strength.

Variables	Males				Females			
	Univariate		Multivariate		Univariate		Multivariate	
	St-β (SE)	*P*-value	St-β (SE)	*P*-value	St-β (SE)	*P*-value	St-β (SE)	*P*-value
**Dep: ALM index**								
Body mass index	0.742 (0.019)	< 0.001	0.694 (0.020)	< 0.001	0.563 (0.020)	< 0.001	0.560 (0.020)	< 0.001
Waist circumference	0.388 (0.010)	< 0.001	0.335 (0.010)	< 0.001	0.162 (0.008)	0.137	0.162 (0.008)	0.142
Thigh circumference	0.755 (0.014)	< 0.001	0.719 (0.018)	< 0.001	0.645 (0.014)	< 0.001	0.616 (0.015)	< 0.001
Arm circumference	0.679 (0.026)	< 0.001	0.631 (0.028)	< 0.001	0.433 (0.023)	< 0.001	0.410 (0.024)	< 0.001
TMC	0.741 (0.015)	< 0.001	0.702 (0.018)	< 0.001	0.711 (0.014)	< 0.001	0.696 (0.017)	< 0.001
MAMC	0.720 (0.030)	< 0.001	0.678 (0.032)	< 0.001	0.544 (0.030)	< 0.001	0.496 (0.033)	< 0.001
**Dep: HGS**								
Body mass index	0.295 (0.186)	0.001	0.197 (0.179)	0.023	–0.100 (0.154)	0.358	–0.091 (0.147)	0.382
Waist circumference	0.318 (0.068)	0.001	0.208 (0.066)	0.018	–0.108 (0.054)	0.321	–0.115 (0.051)	0.268
Thigh circumference	0.352 (0.140)	< 0.001	0.183 (0.149)	0.054	0.189 (0.117)	0.082	0.060 (0.117)	0.578
Arm circumference	0.531 (0.207)	< 0.001	0.404 (0.207)	< 0.001	0.043 (0.169)	0.693	–0.020 (0.161)	0.849
TMC	0.333 (0.146)	< 0.001	0.223 (0.150)	0.017	0.293 (0.127)	0.006	0.181 (0.134)	0.102
MAMC	0.593 (0.237)	< 0.001	0.497 (0.232)	< 0.001	0.189 (0.231)	0.081	0.098 (0.229)	0.356

Multivariate analyses were adjusted for age, presence of diabetes mellitus, weekly Kt/V_urea_, urine volume, serum albumin, C-reactive protein, and use of automated peritoneal dialysis.

Abbreviations: St-β, standardized β; SE, standard error; Dep, dependent variable; ALM, appendicular lean mass; HGS, handgrip strength; MAMC, mid-arm muscle circumference; TMC, thigh muscle circumference.

The logistic regression analyses for predicting sarcopenia are shown in Table 3. In the male patients, all six indices were associated with the prediction of sarcopenia; however, the odds ratio according to a 1-unit increase was the least for the AC and MAMC. In the female patients, all indices, except for the WC, were associated with the prediction of sarcopenia, and the decrease in the odds ratio and statistical significance according to a 1-unit increase was the greatest for the TC and TMC.

**Table 3:** Logistic regression analyses for sarcopenia according to indices.

	Males				Females			
	Univariate		Multivariate		Univariate		Multivariate	
	OR (95% CI)	*P*	OR (95% CI)	*P*	OR (95% CI)	*P*	OR (95% CI)	*P*
Body mass index	0.78 (0.69–0.90)	< 0.001	0.72 (0.60–0.87)	0.001	0.80 (0.67–0.95)	0.011	0.72 (0.57–092)	0.009
Waist circumference	0.95 (0.91–0.99)	0.016	0.94 (0.88–0.99)	0.027	0.99 (0.95–1.04)	0.770	1.00 (0.94–1.06)	0.979
Thigh circumference	0.76 (0.67–0.86)	< 0.001	0.77 (0.66–0.90)	0.001	0.66 (0.53–0.82)	< 0.001	0.64 (0.49–0.82)	< 0.001
Arm circumference	0.66 (0.54–0.81)	< 0.001	0.60 (0.46–0.78)	< 0.001	0.80 (0.66–0.96)	0.019	0.78 (0.62–0.99)	0.039
TMC	0.76 (0.67–0.86)	< 0.001	0.77 (0.66–0.90)	0.001	0.66 (0.53–0.82)	< 0.001	0.64 (0.49–0.82)	< 0.001
MAMC	0.66 (0.54–0.81)	< 0.001	0.60 (0.46–0.78)	< 0.001	0.80 (0.66–0.96)	0.019	0.78 (0.62–0.99)	0.039

Multivariate analysis was adjusted for age, presence of diabetes mellitus, weekly Kt/V_urea_, urine volume, serum albumin, C-reactive protein, and use of automated peritoneal dialysis.

Abbreviations: OR, odds ratio; CI, confidence interval; MAMC, mid-arm muscle circumference; TMC, thigh muscle circumference.

### Predictive value of the six indices for sarcopenia

The AUROC of the indicators of sarcopenia is shown in Table S2. In the male patients, the reciever operating characteristic curve analyses showed that the MAMC and TC predicted sarcopenia better than did the BMI and WC (AC vs. BMI, *P* = 0.061; AC vs. MAMC, *P* = 0.092; AC vs. TC, *P* = 0.633; AC vs. TMC, *P* = 0.818; AC vs. WC, *P* < 0.001; BMI vs. MAMC, *P* = 0.019; BMI vs. TC, *P* = 0.029; BMI vs. TMC, *P* = 0.138; BMI vs. WC, *P* = 0.038; MAMC vs. TC, *P* = 0.720; MAMC vs. TMC, *P* = 0.569; MAMC vs. WC, *P* < 0.001; TC vs. TMC, *P* = 0.660; TC vs. WC, *P* = 0.005; and TMC vs. WC, *P* = 0.020). In the female patients, the TMC, TC, and MAMC predicted sarcopenia better than did the BMI, AC, and WC (AC vs. BMI, *P* = 0.429; AC vs. MAMC, *P* < 0.001; AC vs. TC, *P* = 0.007; AC vs. TMC, *P* = 0.010; AC vs. WC, *P* = 0.248; BMI vs. MAMC, *P* = 0.049; BMI vs. TC, *P* = 0.015; BMI vs. TMC, *P* = 0.015; BMI vs. WC, *P* = 0.139; MAMC vs. TC, *P* = 0.234; MAMC vs. TMC, *P* = 0.140; MAMC vs. WC, *P* = 0.029; TC vs. TMC, *P* = 0.330; TC vs. WC, *P* < 0.001; and TMC vs. WC, *P* < 0.001).

To estimate the incremental value of each index for its association with sarcopenia, we compared the probabilities of events and non-events of the models using the relative IDI and category-free NRI (Table S3). In the male patients, the area under the curve (AUC) value in the model excluding the six indices was 0.78, while those in the models including BMI, WC, TC, AC, TMC, and MAMC were 0.84, 0.82, 0.85, 0.86, 0.86, and 0.88, respectively. The model including the MAMC showed the best AUC value among all models including the covariates and other indices. In the female patients, the AUC value in the model excluding the six indices was 0.76, while those in the models including the BMI, WC, TC, AC, TMC, and MAMC were 0.78, 0.76, 0.86, 0.77, 0.90, and 0.81, respectively. The model including the TMC showed the best AUC value among all models including the covariates and other indices. The results based on the relative IDI and category-free NRI showed trends similar to those from the comparison between the AUC values.

## Discussion

We evaluated the association between the ALM index, HGS, and sarcopenia and the six indices of body measurements using correlation, linear or logistic regression, and AUROC curve analyses. In our study, the prevalence of sarcopenia was 37.7%. A recent meta-analysis showed that the prevalence of sarcopenia in patients receiving PD was 4–48%^5^. Although the prevalence range is very wide due to the different criteria applied for sarcopenia and ethnicities, the prevalence of sarcopenia in our study is within the range reported in previous studies. Our study showed that the circumferences of the lower extremities had the greatest association with the ALM index in both sexes. Prediction of HGS was better with the MAMC than with the other indices in the male patients, whereas none of the indices were associated with HGS in the female patients. Moreover, the MAMC in the male patients and TMC in the female patients were the strongest predictors of sarcopenia among the six anthropometric indices. The TMC was associated with the ALM index; however, HGS as a component of sarcopenia was not associated with the TMC in female patients. The TMC was associated with sarcopenia regardless of HGS. This point should be carefully considered in judging whether the TMC can be applied in screening for sarcopenia in female patients receiving PD.

The association of the ALM index was better with the circumferences of the lower extremities than with the circumferences of the upper extremities, BMI, or WC. The muscle mass of the lower extremities includes a significant amount of appendicular muscle mass, and the lower extremities comprise the primary agonist muscles for exercise and activities of daily life^23^. Therefore, the TMC or TC as an index of the muscle mass of the lower extremities can estimate the ALM index in patients receiving PD. Ohkawa et al. included older women in their study and evaluated the MAMC and thigh muscle area using computed tomography (CT) and creatinine production as indicators of muscle mass^23^. They showed that the thigh muscle area was a better indicator of creatinine production than the MAMC. Kang et al. included patients on maintenance hemodialysis in their study and showed that the thigh muscle area assessed using CT is a better predictor of HGS and physical performance than other muscle indicators^24^. Our results corroborate these previous findings. Moreover, we showed that both TMC and TC performed similarly in predicting the ALM index; however, the association of the TC with the FM of the upper or lower extremities was greater than that of the TMC. Since the TMC excludes the triceps skinfold with subcutaneous FM from the TC, better results with the TMC may be expected.

The calf circumference is a well-known predictor of the muscle mass of the lower extremities or entire body or of sarcopenia. It is useful in evaluating muscle mass considering the large muscle mass and small FM and the easy measurement without the need to undress. Therefore, the calf circumference is currently one of the most commonly used anthropometric measurements for predicting the ALM index or sarcopenia^25^. However, it is affected by edema depending on gravity, and its usefulness is especially limited in volume-dependent patients, such as those on dialysis. We did not evaluate the calf circumference because it was the circumference most influenced by volume. A previous study has shown that patients receiving PD are more prone to developing volume overloading than patients receiving hemodialysis, and this condition would be associated with overestimation of the calf circumference in relation to the gravity effect of volume^26^. Although the proportion of patients with significant pitting edema was not evaluated, we anticipate a high prevalence of pitting edema in the patients receiving PD. In addition, these patients would be prone to developing pretibial pitting edema through the side effect of calcium channel blockers or insufficient venous return by the peritoneal dialysate beyond volume overloading. These inherent risks of pretibial pitting edema can limit the accuracy of the calf circumference. Measurement of this circumference would be applicable after the cessation of calcium channel blockers and strict volume control. However, our observational and cross-sectional study did not perform these interventions before the measurements. Comparisons between various circumferences, including the calf circumference, according to the volume status or presence of pitting edema would be interesting; however, this is beyond the scope of our study.Our study showed that the MAMC was the best predictor of HGS in male patients; however, there were no indices suitable for predicting HGS in female patients. Strength can be influenced by both muscular and neural effects. However, the main factors associated with strength differ between the sexes. In men, muscle mass per se correlates with strength. The MAMC is an indicator of the muscle mass of the upper extremities, and a close association between the MAMC and HGS may be expected. However, women have a limited muscle capacity compared to men, and strength in women is closely influenced by other factors, such as muscle quality or neural factors^27–32^. Body circumferences cannot estimate muscle quality or neural factors. Our analyses showed a non-association between the circumferences of the extremities and HGS in female patients. Assessing the muscle density using CT or electromyography would help identify the muscle quality or neural factors^33,34^.

Among all indices in our study, the MAMC and TMC were the best predictors of sarcopenia in the male and female patients, respectively (Table 3 and Table S2). In male patients, sarcopenia was closely associated with the MAMC. The MAMC was more closely correlated with both the ALM index and HGS than the other indices (Table 2 and Table S1). MAMC measurement as a screening method for predicting sarcopenia would help reduce misclassification of patients with low ALM index and normal HGS or normal ALM index and low HGS as patients with sarcopenia. If an indicator is closely associated with only the ALM index, it will misclassify patients with low ALM index and normal HGS as patients with sarcopenia. This would lead to a large proportion of misclassified patients as compared with a classification using an indicator closely associated with both ALM index and HGS. In female patients, the TMC showed the best association with the ALM index; however, no indices were associated with HGS. These possibilities for misclassification should be considered when the TMC is used to screen for sarcopenia in female patients.

Patients receiving dialysis are prone to exposure to abnormal nutritional, metabolic, and inflammatory environments, which are associated with an accelerated development of sarcopenia^5^. Sarcopenia in these patients has been an important topic after the publication of the sarcopenia consensus. Sarcopenia in patients receiving dialysis is not completely identical in pathophysiology to that in the general population^2^. In addition, the application of diagnostic measurements is limited in patients receiving dialysis owing to volume overloading and differences in the distribution of body composition. Therefore, further studies including patients receiving dialysis with a mechanism or nature different from that of the general population, would help understand the usefulness of diagnostic measurements or pathophysiology of sarcopenia in patients with various comorbidities. Body circumferences, including those of the extremities, can be easily calculated. Previous studies in patients receiving dialysis have evaluated the usefulness of body circumferences in predicting sarcopenia. Noori et al. evaluated patients receiving hemodialysis and showed that the MAMC is associated with LM based on DXA or near-infrared interactance findings^35^. Bataille et al. enrolled patients receiving hemodialysis and showed the predictability of sarcopenia using various measurements, including the muscle mass index, BMI, HGS, AC, and TC^36^. They showed a positive association between sarcopenia and the AC in both sexes and the TC in men. Sai et al. compared the TC with a sonographic assessment of the quadriceps muscle in patients receiving hemodialysis^37^. Although sonographic assessment of the quadriceps muscle was superior to the TC in predicting sarcopenia, the TC was associated with fall risk or patient survival in men. Two studies enrolled patients with chronic kidney disease of various stages and patients receiving PD and revealed the usefulness of the calf circumference in predicting sarcopenia or low muscle mass^38,39^. These studies evaluated the association between sarcopenia and body circumferences; however, comparisons between various appendicular circumferences or subgroup analyses by sex have not been fully conducted.

Adjustment of LM is an important issue. In our study, the ALM index was adjusted based on the height squared. Although the height adjusted ALM index is a commonly employed parameter for predicting relative muscle mass in relation to body size, height-adjusted mass can underestimate the prevalence of low muscle mass, especially in patients with high FM or obesity^40–44^. Newman et al. suggested adjustment for both height and FM to overcome underestimation of sarcopenia in patients with obesity and reported favorable results compared with those after adjustment of height alone^40^. Therefore, the Foundation for the National Institutes of Health Sarcopenia Project suggested the use of BMI-adjusted muscle mass for the diagnosis of low muscle mass^41^. Previous studies in the general population and patients receiving hemodialysis showed that the BMI adjustment method is better for predicting muscle strength, frailty, physical performance, activities of daily living performance, or cardiometabolic syndrome than the height adjustment method^42–44^. However, there are few data regarding the optimal adjustment for predicting strength or adverse outcomes in patients receiving PD. Further studies are needed to identify better adjustment methods for these patients.

This study has several limitations. First, the study was a single-center retrospective study. Second, it was limited by its small sample size. Additionally, the sample was divided into two groups according to sex. The small sample size limited full adjustment for covariates and subgroup analyses according to various characteristics. Third, our study included measurement of the ALM index, HGS, and six indices at a single time-point without follow-up data and did not include some important variables, such as physical performance, which may be better indicators than just sarcopenia alone. Prospective studies using larger samples, data with repeated and follow-up measurements, and data on physical performance are needed to overcome these limitations.

In conclusion, this study demonstrated that the circumferences of the lower extremities might be the best predictors of the ALM index in both sexes in PD patients. The MAMC might be the best predictor of HGS in male PD patients. The MAMC in male PD patients and TMC in female PD patients might be the best predictors of sarcopenia. However, the TMC was associated with sarcopenia regardless of HGS in the female PD patients. These findings suggest that different indices should be considered in predicting sarcopenia or its components based on the sex in PD patients.

## Supplementary Information

Supplementary Information 1.

Supplementary Information 2.

Supplementary Information 3.

## Data Availability

All data relevant to the study are included in the article or uploaded as supplementary information.
