# Supplementary material for: Comparison of various indices for predicting sarcopenia and its components in patients receiving peritoneal dialysis
Source: Sci Rep. 2022 Aug 18;12:14102. doi: 10.1038/s41598-022-18492-2 (PMC9388491; doi:10.1038/s41598-022-18492-2)
Supplement: Supplementary file 1 — Supplementary Information 1. [file 41598_2022_18492_MOESM1_ESM.doc]

**Table S1. Correlation between the indices and the fat mass and lean mass**

|  | **Arms FM** | **Arms LM** | **Legs FM** | **Legs LM** | **ALM index** | **HGS** |
| --- | --- | --- | --- | --- | --- | --- |
| **Univariate** |  |  |  |  |  |  |
| Male |  |  |  |  |  |  |
| Body mass index | 0.797* | 0.936* | 0.702* | 0.622* | 0.742* | 0.295^#^ |
| Waist circumference | 0.793* | 0.501* | 0.772* | 0.328* | 0.388* | 0.318^#^ |
| Thigh circumference | 0.623* | 0.656* | 0.596* | 0.782* | 0.755* | 0.352* |
| Arm circumference | 0.701* | 0.738* | 0.673* | 0.613* | 0.679* | 0.531* |
| TMC | 0.394* | 0.643* | 0.389* | 0.825* | 0.741* | 0.333* |
| MAMC | 0.493* | 0.793* | 0.493* | 0.714* | 0.720* | 0.593* |
| Female |  |  |  |  |  |  |
| Body mass index | 0.407* | 0.374* | 0.261^#^ | 0.334^#^ | 0.563* | –0.100 |
| Waist circumference | 0.417* | 0.252^#^ | 0.263^#^ | 0.136 | 0.162 | –0.108 |
| Thigh circumference | 0.288^#^ | 0.394* | 0.204^#^ | 0.631* | 0.645* | 0.189 |
| Arm circumference | 0.406* | 0.375* | 0.292^#^ | 0.328^#^ | 0.433* | 0.043 |
| TMC | 0.081 | 0.446* | 0.107 | 0.757* | 0.711* | 0.293^#^ |
| MAMC | 0.304 | 0.495* | 0.271^#^ | 0.492* | 0.544* | 0.189 |
| **Multivariate** |  |  |  |  |  |  |
| Male |  |  |  |  |  |  |
| Body mass index | 0.775* | 0.589* | 0.653* | 0.580* | 0.618* | 0.222^#^ |
| Waist circumference | 0.768* | 0.464* | 0.730* | 0.280^#^ | 0.343* | 0.232^#^ |
| Thigh circumference | 0.595* | 0.519* | 0.533* | 0.714* | 0.681* | 0.190 |
| Arm circumference | 0.665* | 0.687* | 0.604* | 0.561* | 0.638* | 0.447* |
| TMC | 0.346* | 0.535* | 0.315^#^ | 0.779* | 0.675* | 0.234^#^ |
| MAMC | 0.452* | 0.758* | 0.419* | 0.678* | 0.685* | 0.549* |
| Female |  |  |  |  |  |  |
| Body mass index | 0.400* | 0.344^#^ | 0.275^#^ | 0.358^#^ | 0.574^#^ | –0.100 |
| Waist circumference | 0.409* | 0.218 | 0.261^#^ | 0.184 | 0.167 | –0.126 |
| Thigh circumference | 0.263^#^ | 0.315^#^ | 0.138 | 0.565* | 0.616* | 0.064 |
| Arm circumference | 0.389^#^ | 0.305^#^ | 0.270^#^ | 0.326^#^ | 0.421* | –0.022 |
| TMC | 0.054 | 0.369^#^ | 0.044 | 0.681* | 0.667* | 0.185 |
| MAMC | 0.285^#^ | 0.401* | 0.243^#^ | 0.456* | 0.498* | 0.105 |

Data are expressed as correlation coefficients. Correlation coefficients were calculated using Pearson’s correlation for univariate and partial correlation for multivariate analyses. Partial correlation was adjusted for age, presence of diabetes mellitus, weekly Kt/V_urea_, urine volume, serum albumin, C-reactive protein, and use of automated peritoneal dialysis. *P < 0.001; ^#^P < 0.05.

Abbreviations: FM, fat mass; LM, lean mass; ALM, appendicular lean mass; HGS, handgrip strength; MAMC, mid-arm muscle circumference; TMC, thigh muscle circumference.
