# Supplementary material for: Comparison of various indices for predicting sarcopenia and its components in patients receiving peritoneal dialysis
Source: Sci Rep. 2022 Aug 18;12:14102. doi: 10.1038/s41598-022-18492-2 (PMC9388491; doi:10.1038/s41598-022-18492-2)
Supplement: Supplementary file 2 — Supplementary Information 2. [file 41598_2022_18492_MOESM2_ESM.doc]

**Table S2. Area under the curves for prediction of sarcopenia by various indices**

|  | **AUC (95% CI)** | ***P*-value** |
| --- | --- | --- |
| Males |  |  |
| BMI | 0.71 (0.62–0.79) | <0.001 |
| WC | 0.62 (0.52–0.71) | 0.244 |
| TC | 0.79 (0.70–0.86) | <0.001 |
| AC | 0.77 (0.68–0.84) | <0.001 |
| TMC | 0.78 (0.69–0.85) | <0.001 |
| MAMC | 0.81 (0.72–0.87) | <0.001 |
| Females |  |  |
| BMI | 0.68 (0.57–0.77) | 0.003 |
| WC | 0.51 (0.40–0.62) | 0.899 |
| TC | 0.82 (0.72–0.89) | <0.001 |
| AC | 0.65 (0.54–0.75) | 0.014 |
| TMC | 0.84 (0.75–0.91) | <0.001 |
| MAMC | 0.75 (0.65–0.84) | <0.001 |

Abbreviations: AUC, area under the curve; CI, confidence interval; BMI, body mass index; WC, waist circumference; TC, thigh circumference; AC, arm circumference; TMC, thigh muscle circumference; MAMC, mid-arm muscle circumference.
