# Supplementary material for: Comparison of various indices for predicting sarcopenia and its components in patients receiving peritoneal dialysis
Source: Sci Rep. 2022 Aug 18;12:14102. doi: 10.1038/s41598-022-18492-2 (PMC9388491; doi:10.1038/s41598-022-18492-2)
Supplement: Supplementary file 3 — Supplementary Information 3. [file 41598_2022_18492_MOESM3_ESM.doc]

**Table S3. Area under the curves, integrated discrimination improvement, and net reclassification improvement for multivariate models with or without sarcopenia.**

| **Models** | **AUC** | **Difference between AUCs** | | **Relative IDI** | | **Category-Free NRI** | |
| --- | --- | --- | --- | --- | --- | --- | --- |
|  |  | **Values** | ***P*-value** | **Values** | ***P*-value** | **Values** | ***P*-value** |
| **Males** |  |  |  |  |  |  |  |
| Multivariate model | 0.78 |  |  | – | – | – | – |
| Multivariate model with BMI | 0.84 | 0.06 | 0.054 | 0.47 | <0.001 | 0.78 | <0.001 |
| Multivariate model with WC | 0.82 | 0.04 | 0.162 | 0.18 | 0.017 | 0.36 | 0.065 |
| Multivariate model with TC | 0.85 | 0.07 | 0.033 | 0.46 | <0.001 | 0.85 | <0.001 |
| Multivariate model with AC | 0.86 | 0.08 | 0.018 | 0.64 | <0.001 | 0.76 | <0.001 |
| Multivariate model with TMC | 0.86 | 0.08 | 0.017 | 0.54 | <0.001 | 0.76 | <0.001 |
| Multivariate model with MAMC | 0.88 | 0.09 | 0.008 | 0.84 | <0.001 | 1.03 | <0.001 |
| **Females** |  |  |  |  |  |  |  |
| Multivariate model | 0.76 |  |  | – | – | – | – |
| Multivariate model with BMI | 0.78 | 0.03 | 0.437 | 0.28 | 0.044 | 0.40 | 0.082 |
| Multivariate model with WC | 0.76 | 0.00 | 0.480 | 0.00 | 0.988 | –0.12 | 0.603 |
| Multivariate model with TC | 0.86 | 0.10 | 0.064 | 0.69 | <0.001 | 0.84 | <0.001 |
| Multivariate model with AC | 0.77 | 0.01 | 0.647 | 0.14 | 0.175 | 0.29 | 0.196 |
| Multivariate model with TMC | 0.90 | 0.14 | 0.022 | 1.19 | <0.001 | 1.06 | <0.001 |
| Multivariate model with MAMC | 0.81 | 0.06 | 0.055 | 0.46 | 0.004 | 0.57 | 0.012 |

Dependent variable was sarcopenia, and the multivariate model used age, presence of diabetes mellitus, weekly Kt/V_urea_, urine volume, serum albumin, C-reactive protein, and use of automated peritoneal dialysis.

**Abbreviations**: AUC, area under the curve; IDI, integrated discrimination improvement; NRI, net reclassification improvement; BMI, body mass index; WC, waist circumference; TC, thigh circumference; AC, arm circumference; TMC, thigh muscle circumference; MAMC, mid-arm muscle circumference.
